# Multidimensional interventions to increase life-space mobility in older adults ranging from nursing home residents to community-dwelling: a systematic scoping review

**DOI:** 10.1186/s12877-023-04118-3

**Published:** 2023-07-06

**Authors:** Julia Seinsche, Carl-Philipp Jansen, Sandro Roth, Wiebren Zijlstra, Timo Hinrichs, Eleftheria Giannouli

**Affiliations:** 1 Department of Health Sciences & Technology, Institute of Human Movement Sciences & Sport, ETH Zurich, Zurich, Switzerland; 2 Robert-Bosch-Krankenkaus, Stuttgart, Germany; 3 Division of Sports and Exercise Medicine, Department of Sport, Exercise, and Health, University of Basel, Basel, Switzerland; 4 Institute of Movement & Sport Gerontology, German Sport University Cologne, Cologne, Germany

**Keywords:** Real-life mobility, Multidimensional interventions, Life-Space Assessment

## Abstract

**Background:**

Life-space mobility (LSM) is an important aspect of older adults’ real-life mobility. Studies have shown that restricted LSM is a risk factor for many adverse outcomes such as low quality of life and mortality. Therefore, an increasing number of interventions aim to enhance LSM. However, the intervention approaches differ in terms of their type/content, duration, targeted populations, but also in terms of their outcome measures and assessment tools. Especially the latter impairs the comparability of studies with otherwise similar interventional approaches and thus also the interpretation of their results. Therefore, this systematic scoping review aims to provide an overview of the intervention components, assessment tools, and effectiveness of studies aiming to improve LSM in older adults.

**Methods:**

A systematic literature search was carried out in PubMed and Web of Science. We considered studies in older adults of any design that included an intervention approach and at least one outcome of LSM.

**Results:**

27 studies were included in the review. These studies analyzed healthy community-dwelling as well as frail older adults in need of care or rehabilitation and nursing home residents with a mean age between 64 and 89. The percentage of female participants ranged from 3 to 100%. The types of interventions were of the following: physical, counseling, multidimensional, miscellaneous. Multidimensional interventions consisting of physical interventions plus any of the following or a combination of counseling/education/motivation/information appear to be most effective in increasing LSM. Older adults with mobility impairments were more responsive to these multidimensional interventions compared to healthy older adults. Most of the studies used the questionnaire-based Life-Space Assessment to quantify LSM.

**Conclusions:**

This systematic scoping review provides a comprehensive overview of a heterogenous stock of literature investigating LSM-related interventions in older adults. Future meta-analyses are needed to provide a quantitative evaluation of the effectiveness of LSM interventions and recommendations.

**Supplementary Information:**

The online version contains supplementary material available at 10.1186/s12877-023-04118-3.

## Background and objectives

Life-Space Mobility (LSM) is an important aspect of a person’s real-life mobility [1, 2]. Unlike mobility capacity and motor function, LSM is a more complex multifactorial construct [3], which addresses a person’s physical as well as social environment [4]. Therefore, it can be described as in- or out-of-home mobility defined by the space in which a person moves in her or his daily life, respectively by the distance of these spaces from home [3, 5, 6]. Assessment instruments to quantify life-space share that they depict various life-space zones spanning from an individual’s bedroom or home to places beyond an individual’s home town or even further [6].

Assessing LSM has proven to be highly relevant for many populations, especially for older adults. Before COVID, 12-13.5% of male and 27.1% of female older adults were found to have severely impaired LSM [7, 8], and during COVID, this number increased significantly [9]. A restricted life-space has been associated with higher subsequent healthcare costs and higher risks of subsequent submission in hospital or nursing care [10]. Thus, in older adults with difficulties walking ¼ mile, total annual healthcare costs have increased about $2773 [11]. What is more, it was shown that restricted LSM is a risk factor for numerous adverse health-related factors in older adults like poor physical health and functioning [12], cognitive impairment [7, 8, 13, 14], low quality of life [15, 16], restricted social participation [16, 17], nursing home admission [18], loss of independence and increased risk of mortality [17, 19–21]. What is more, a number of studies have identified several factors that can influence life-space mobility in older adults [22–28]. Webber et al. [3] categorized those influencing factors into five categories: (1) physical abilities, (2) cognitive abilities, (3) psychological factors, (4) environmental factors, and (5) financial factors with gender, culture and biography affecting each category. Identifying factors that can influence LSM is important because they can in turn serve as targets for the development of interventions to increase LSM or prevent age-related decline in LSM.

In the last years there has been an increase in the number of studies aiming to enhance LSM. Due to the complexity and multidisciplinary nature of factors influencing LSM, intervention approaches differ in terms of their type/content (e.g., exercise interventions targeting functional capacity versus consultations/behavioral modification techniques), duration (one-time session to regular sessions for months), targeted populations (e.g., healthy, frail, cognitively-impaired), but also in terms of their outcome measures and assessment tools (e.g., self-reported questionnaires, objective GPS-based tools). Especially the latter impairs the comparability of studies with otherwise similar/same interventional approaches and thus also the interpretation of their results.

Therefore, we aimed to provide a comprehensive overview of a potentially large and heterogenous stock of literature investigating LSM-related interventions in older adults. Consequently, this systematic scoping review investigated the following research questions:

1. Which intervention components are used to increase life-space mobility in older adults?

2. Which assessment tools are used to measure life-space mobility in intervention studies in older adults?

3. How effective are those interventions to increase life-space mobility in older adults?

### Research design and methods

Scoping reviews have been defined as a “valuable resource for informing future systematic reviews (…) can be of use to researchers, policy-makers and practitioners, reducing duplication of effort and guiding future research” [29]. Correspondingly, we chose to conduct a scoping review to demonstrate the large variety of study designs of LSM-related intervention studies, the divers study populations, intervention methods, and outcome parameters and measures. Yet, we also aimed to cluster interventions and synthesize findings with respect to their effectiveness in order to identify the possible scope for a more precise systematic review and to define boundaries such as the definition of appropriate intervention types and outcomes and more specific research questions.

### Protocol and registration

This systematic scoping review was conducted according to the PRISMA guidelines’ extension for scoping reviews (PRISMA-ScR, Supplementary Table 1, Additional File 1) [30], and registered on Prospero (registration ID: CRD42021236380) on March 27th 2021.

### Search strategy

In February 2021 and May 2023, PubMed and Web of Science were systematically searched using a combination of keywords describing LSM and its synonyms, a variety of terms to describe intervention types, and various expressions to define the target population. For PubMed, this resulted in a search strategy presented in Table 1.

Besides that, a hand-search was conducted in google scholar and other studies known to the authors were screened to ensure literature saturation.

**Table 1:** Search Strategy for PubMed

Population	Intervention	Outcome of interest
#21 “older adult*” [tiab]^a^ #22 “older person*” [tiab] #23 elder* [tiab] #24 elderly [tiab] #25 seniors [tiab] #26 “nursing home” [tiab] #27 “nursing homes” [tiab] #28 “care home” [tiab] #29 “care homes” [tiab] #30 “assisted living” [tiab] #31 Aged [Mesh]^b^ #32 “Aged, 80 and over” [Mesh] #33 aging [Mesh] #34 “long-term care” [Mesh] #35 “nursing homes” [Mesh] #36 “homes for the aged” [Mesh] #37 “assisted living facilities” [Mesh] #38 OR (#21-#37) #39 (#8 AND #20 AND #38)	#9 intervent* [tiab] #10 intervention [tiab] #11 exercis* [tiab] #12 exercise [tiab] #13 rehab* [tiab] #14 rehabilitation [tiab] #15 therap* [tiab] #16 therapy [tiab] #17 training [tiab] #18 prevention [tiab] #19 program* [tiab] #20 OR (#9-#19)	#1 “life-space” [tiab] #2 lifespace [tiab] #3 “life space” [tiab] #4 “real-life mobility” [tiab] #5 “out-of-home” [tiab] #6 “global positioning system” [tiab] #7 “life zone” [tiab] #8 OR (#1-#7)

*Note.*^a^ tiab: Title or Abstract; ^b^ Mesh: Medical subject headings

### Selection criteria

Inclusion criteria were (1) articles published in English, (2) study population consisting of older adults aged 60 years or older, (3) the study was conducted as an intervention study including a pre-post assessment, (4) outcomes explicitly included LSM - either subjectively determined (that is via questionnaires such as the University of Alabama Life-Space Assessment (LSA)) or objectively (e.g., via GPS). Studies focusing solely on physical activity-related outcomes (e.g., walking times) and articles published as registries, conference abstracts or editorials were excluded. As recommended for scoping reviews [29], inclusion and exclusion criteria were rather broad and study quality was not used as a filter in order to map all existing literature. Accordingly, there were no restrictions regarding health condition, type of interventions, control group characteristics or year of publication.

### Study selection and organization

The resulting articles of both databases were imported into Mendeley citation manager, duplicates were removed. Subsequently, the remaining articles were imported into Rayyan [31], where in a first step, two reviewers (JS and SR) independently screened titles and abstracts. After discussing the results, the same two reviewers conducted a blinded full-text screening to evaluate the remaining studies in more detail. A third and, if necessary, a fourth reviewer (EG and CPJ) were consulted in case of any disagreements after both screening steps; decisions were discussed until consensus was reached.

To organize the articles, included studies were labelled using pre-specified terms relating to the following categories: (1) assessment tool to measure LSM, (2) intervention type, (3) population characteristics. Reasons for exclusion were also indicated using pre-defined labels provided by Rayyan in the following order: (1) wrong language, (2) wrong outcome, (3) wrong population, (4) wrong study design, and (5) wrong publication type. The first label of this order which was found to be applicable was indicated as reason for exclusion.

### Data extraction and analysis

Next to the labels described above, more detailed information (number of participants, study design, intervention details, timeframe, other outcome measures, and results) was extracted and transferred to an excel spreadsheet. Details can be found in Supplementary Table 2 (Additional File 2). In case only study protocols were available, the authors were asked to provide the missing information to fill in the extraction table.

### Risk of bias and quality assessment

Most commonly, scoping reviews do not include an assessment of the methodological quality of included studies, however, the lack of quality assessment is often mentioned as a limitation factor of scoping reviews [32]. Consequently, we incorporated a critical appraisal of included studies in order to overcome this limitation of scoping reviews and to provide guidance for treatments or future reviews. The “NIH Quality Assessment Tool for Controlled Intervention Studies”, respectively the “NIH Quality Assessment Tool for Before-After (Pre-Post) Studies with No Control Group” were used [33]. The former includes items about randomization, blinding, similarity of groups at baseline, dropouts, adherence, outcome measures, power calculation, and type of analyses. Items of the latter tool comprise questions about the study question, eligibility criteria, study population, sample size, description of interventions, outcome measures, blinding, follow-up rate, statistical analyses, and group-level interventions. Concerning controlled intervention studies, each study was given a score out of 14 based on the number of “yes” given to each question. A score of 10–14 indicated a good, 5–9 a fair, and 0–4 a low quality. Regarding pre-post studies, the total score was 12 and 8–12 points meant a good quality, whereas 4–7 points indicated a fair, and 0–3 points a low quality.

### Data synthesis

Results were grouped regarding the type of intervention and/or sample characteristics and/or outcome measures and/or time frame and/or study quality to form a clear descriptive summary of the included studies for specific intervention types and target groups. Subsequently, we performed a narrative synthesis to summarize the results.

## Results

**Fig. 1:**
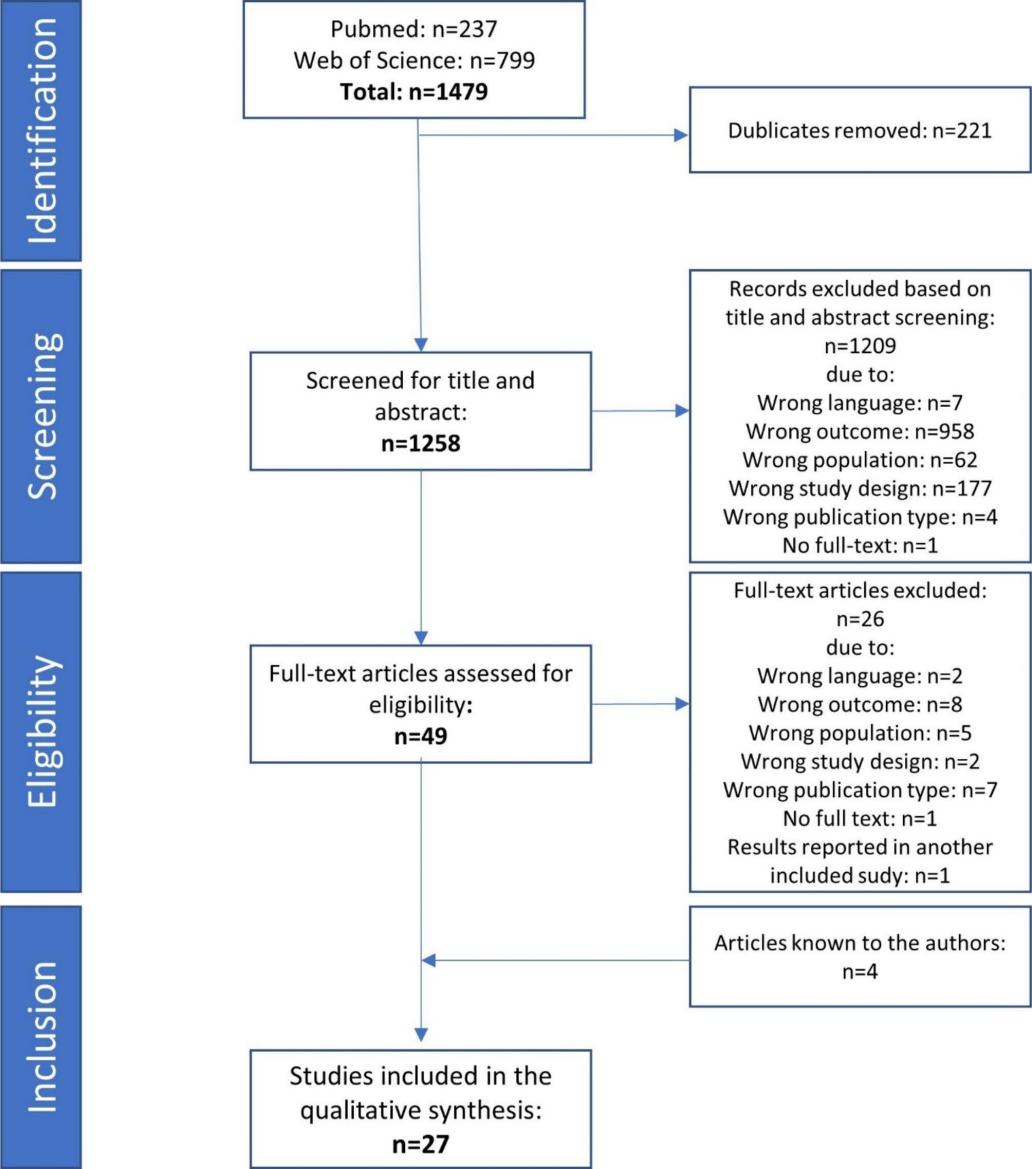
Flow diagram of the systematic literature review

The search strategy resulted in 1479 articles including 221 duplicates, that is, n = 1258 articles were screened for title and abstract. Of these, 49 full texts were analyzed of which 23 met the inclusion criteria. Three further studies were only available as study protocols and have not to date been published as result papers which is why they are among the excluded studies. Finally, 4 further relevant articles [34–37] known to the authors were included. The final sample consisted of n = 27 articles (see Fig. 1). Details of each included study can be found in Supplementary Table 2 (Additional file 2).

### Study characteristics and quality assessment

The 27 included studies were published between 2004 [38] and 2022 [37, 39] and most of them (n = 16) were randomized controlled studies (RCT). Only three studies were conducted as non-randomized controlled trials [37, 40, 41], one study as quasi-randomized [42], and seven further studies as pre-post intervention studies without a control group [35, 36, 38, 39, 43–45].

According to the quality assessment conducted via NIH Quality Assessment Tools, most included studies showed fair (n = 11) to good (n = 14) quality and only two studies demonstrated a poor study quality (Supplementary Tables 3 and 4, Additional File 3).

### Intervention characteristics

Four groups of interventions have been identified which are described in the following. An overview is provided in Supplementary Table 5 (Additional File 4).

### Multidimensional interventions

Almost half of the included studies (n = 12) applied multidimensional interventions, which mostly consisted of physical interventions combined with counseling (n = 10). Additionally, some of these ten studies also included care training/competence training of staff members and/or relatives (n = 2) [41, 46] or community improvements (n = 1) [34]. In most of these multidimensional interventions, the physical part was conducted as a physiotherapy treatment [35, 36, 46] or otherwise as walking interventions [34, 38, 47], mixed physical interventions [37, 41, 48, 49]. The counseling part mostly comprised the encouragement for physical activity and participation [34, 35, 41, 47], nutrition assessment/counseling [37, 38, 46, 49], motivational strategies [48] or ADL-techniques [36]. The remaining two studies which analyzed a multicomponent intervention applied occupational therapy combined with required repairs and home modifications by a handyman [50], or investigated a cognitive-motor group-based activity program [42].

Apart from three studies [37, 38, 42], all multicomponent interventions demonstrated significant positive effects on life-space mobility compared to the respective control group and/or in pre-post-intervention comparison.

### Physical interventions

Six studies [40, 44, 45, 51–53] investigated the effects of purely physical exercise-based interventions on LSM in older adults. These comprised multifaceted walking events/walking tasks [40], gait training [51], outdoor aerobic training [51], and physical exercises (strength, balance, postural exercises, walking, individual instruction, home-based program) [44, 45, 52, 53]. Only three of these studies [40, 44, 45] demonstrated significant between-, respectively within-group effects of physical interventions on LSM in older adults.

### Counseling interventions

Four counseling, respectively educational or psychological interventions were conducted as well. The “Lifestyle Redesign” for instance is an occupational therapy intervention consisting of group sessions and individual meetings and is supposed to empower older adults to perform healthy and fulfilling activities. Levasseur et al. [43] investigated its effect on life-space mobility. They could show an increase in LSM between two out of four assessment points. Nonetheless, no significant improvement was detected over the whole study period. Further studies like Kamga et al. [54], who analyzed the influence of self-care tools such as audio tools and a notebook with written tools together with a coaching, and Siltanen et al. [55], who aimed at increasing self-selected out-of-home activities by counseling, did not find significant effects at all. In contrast, Uemura et al. [56] performed an active learning program regarding behavioral changes to promote a healthy lifestyle which they found to have a significant impact on life-space mobility.

### Miscellaneous interventions

A final group of interventions (n = 6) dealt with very specific and special interventions. These combined breathing therapy with exercise advice, a handheld fan and a calming hand [57], or analyzed a driving cessation program [58], a rise-assisting robot [39], music therapy [59], a wheelchair adaption [60], and a horticulture activity program compared to a multicomponent physical exercise program (described in the section “physical interventions”) [53]. Only the first three types of interventions showed significant effects, while neither of the last three studies improved life-space mobility.

### Sample characteristics

Together, the 27 studies included 2593 participants. The mean age of the populations under investigation ranged from 64 [38] to 89 [60], whereas the proportion of female participants varied from 3% [47] to 100% [44] with only four studies with male predominance [35, 42, 47, 57]. The sample sizes ranged from 3 [39] to 305 [34] participants. Four groups of participants have been identified:

### Community-dwelling older adults without functional limitations

Seven studies investigated LSM in community-dwelling older adults without any specific functional limitations [34, 38, 45, 51, 55, 56, 58]. LSM improved in some of these studies in which a multidimensional intervention [34], counseling [56], or a driving program [58] were conducted. Purely physical exercises did not show an effect on LSM in healthy older adults [45, 51].

### Community-dwelling older adults with functional limitations

Seven studies included community-dwelling older adults categorized as prefrail [37] or frail [45, 49, 59], respectively with disabilities [43, 44, 50]. Thereby, five showed an increase in LSM due to multidimensional interventions [50, 61], physical exercises [44, 45], or counseling [43].

Among this population group, two studies focused on older adults undergoing home-based rehabilitation [35, 36]. In both studies, a multidimensional intervention led to an increased LSM.

Finally, a multidimensional intervention also had a positive impact in cognitively impaired older adults recently discharged from rehabilitation [48].

### Nursing home residents

Six studies included nursing home-residents, respectively older adults receiving nursing care [39, 41, 42, 46, 52, 60]. In three of these studies, LSM was improved by multidimensional interventions [41, 46], or by providing a rise-assisting robot [39].

### Various patient populations

Two studies [47, 57] analyzed internal medicine patients, one study orthopedic patients [40], and depressive participants/participants with psychological problems were in the focus of two studies [53, 54]. The former two groups benefited from a multidimensional intervention [47], a walking intervention [40] and a breathing therapy [57], whereas in depressive participants neither physical interventions, nor counseling or horticulture improved LSM.

### Timeframe

The length of the intervention period ranged from one single event of 90 min [40], to 12 months including 10 times 45 to 60-minute sessions and 3–5 times/week independent training [49]. However, no effect of length and frequency of the interventions on LSM was apparent since both short [40, 54] and long intervention periods [49, 55] showed positive as well as negative results.

### LSM measures

The majority of studies (n = 18) used the Life-Space Assessment (LSA) to quantify the participants’ LSM [35–38, 40, 43–45, 47–49, 51–56, 59], whereas three studies applied the Nursing Home Life-Space Diameter (NHLSD) [42, 46, 60]. Other measurement tools were the Homebound Mobility Assessment (HBMA) [50], the Life-Space Questionnaire (LSQ) [57], GPS (Global Positioning System) and accelerometer [34], an indoor wireless sensor network used to measure the life space of nursing home residents [41], and a single question [39, 58].

LSM was the primary outcome in eighteen studies. Fifteen of these studies demonstrated a positive effect of the respective intervention on LSM [34–36, 39–41, 44–50, 57, 58]. In contrast, of the other nine studies that analyzed LSM as a secondary outcome, only one study indicated a significantly positive effect [56].

## Discussion and implications

### Research questions

The aim of this systematic scoping review was to create a comprehensive overview of the current status of interventions targeting LSM in older adults. Given that LSM is a rather holistic measure of mobility and related to a large number of beneficial health outcomes, this review is helpful to shed light on a currently growing body of research which is of high clinical value to the older population. Against this background, three research questions were posed regarding intervention components, assessment tools, and intervention effects.

Regarding the interventions’ components, a wide range of approaches has been applied, whereby a large number of studies used a multidimensional approach mostly based on a combination of physical exercises and counseling. Physical exercise interventions, comprising strength training, balance training, and walking activities, followed by counseling interventions alone formed the other two major categories. The latter ones were without clear focus on LSM but mainly directed at activity encouragement, nutrition, and motivation. Finally, a last group of miscellaneous studies conducted very specific interventions targeted at equally specific populations, like a hand-held fan for patients with respiratory diseases, novel wheelchairs, rise-assisting robots, or horticulture programs.

To measure the effects of the interventions, apart from GPS-based analyzes, all but two studies used validated questionnaires such as the LSA.

Regarding the interventions’ effectiveness, the included studies revealed many differences which were based on several factors like intervention type, study quality, sample characteristics, and assessment instruments which will be discussed in the following.

### Intervention type

LSM is a complex construct. According to previous studies, a large number of interdisciplinary factors affect life-space mobility in older adults [62–65]. These factors range from physical and cognitive abilities, over various psychological factors like fear of falling and self-efficacy, to diverse environmental factors. Thus, a single-dimension intervention approach does not seem to be appropriate to address the multifaceted nature of LSM. Thereby, it appears that the integration of physical exercises/physiotherapy is essential. This is consistent with other studies that have found physical factors to be most, but not solely associated with LSM [2, 12] and in agreement with this review’s findings that multidisciplinary approaches seem to be the most effective to increase LSM.

This positive effect of multidimensional interventions might, however, be dependent on the participants’ cognitive status. Tanaka et al. [42] analyzed people suffering from dementia who were apparently less responsive to such treatments. This is also indicated by Shaw et al. [66] and by “The American Geriatrics Society/British Society (AGS/BGS) guideline” [67], which does recommend multifactorial interventions, but regarding the components of the intervention, it clearly differentiates between community-dwelling older people, persons in long-term care, and persons in acute hospital settings.

Furthermore, differences were detected among the mono-factorial approaches, especially concerning physical exercise interventions. Studies like Matsuda et al. [44] and Nakagawa et al. [45] applied more complex physical exercise training, (i.e., a combination of muscle strengthening, balance training and/or stretching exercises and/or different walking tasks) and – unlike simpler physical activity programs [51, 52] - demonstrated significant effects on LSM. So again, multicomponent approaches targeting various physical skills showed more positive results emphasizing the complex physical challenges everyday mobility poses on older adults.

A systematic review by Ross et al. [68] attempted to investigate all possible interventions to maintain mobility, whereby they defined mobility as “any objective or self-report measure of every activity as it relates to the purposeful movement of an individual through physical space” – a definition which encompasses but does not focus on life-space mobility. Most importantly, similar to the findings of the current review, they found mixed results of physical activity intervention studies, but positive outcomes of multicomponent training interventions.

The same applied to purely counseling interventions. Two studies [43, 56] conducted interventions involving multiple components like group sessions, self-planning and counseling, which led to mixed, respectively positive results. On the contrary, interventions mainly based on only self-care tools [54] or phone calls plus supportive materials [55] did not show any effects. This matches again with the review of Ross et al. [68] who found mixed results concerning the effect of educational interventions on several mobility outcomes. They suggest that interventions tailored to specific participant characteristics are more likely to be effective. Another point to consider is obviously the social aspect and the group effect, which have proven to have a positive influence on adherence [69, 70].

Interventions designed for very specific target groups which involved wheelchair adaptions [60], a horticultural program [53], and music therapy [59] did not show significant effects on LSM. This indicates again that these specific interventions are not able to cover all relevant aspects of the complexity of LSM, especially considering that these topics do not or only vaguely touch LSM.

Correspondingly, a very strongly distinguishing factor is the assignment of LSM as either primary or secondary outcome of the respective study. It is striking that the vast majority of the studies analyzing LSM as primary outcome showed positive effects, whereas studies which treated LSM as a secondary outcome did not. The interventions of the latter group were not primarily designed for an improvement of LSM and were consequently too unspecific.

### Study quality

Only seven studies [36, 45–49, 56] revealed both, a “good” quality and at the same time significant improvements in LSM. It is striking that all but two [45, 56] of these seven studies applied multidimensional treatments and that apart from Uemura et al. [56], all of them analyzed LSM as a primary outcome. Due to their good quality, these studies can be considered potentially the most effective and meaningful intervention approaches. Thus, the special role of multidimensional and outcome specific interventions is even more highlighted. Nevertheless, the number of good-quality studies is still too low to draw robust conclusions about statements regarding suitable interventions to increase LSM in older people.

### Time frame

The duration of the intervention period did not seem to have an influence on LSM. Studies with both shorter (4 weeks) [46] and longer (12 month) [49] intervention periods brought forth significant improvements in LSM, which indicates that the intervention type rather than the duration of the intervention determines the older adults’ adherence to the programs and their responsiveness.

However, regardless of the duration of the intervention period and regardless of other factors like the type of intervention, the sustainability of positive outcomes is an occurring issue which needs to be addressed [41, 46, 48, 58]. For instance, a continuous therapeutical intervention program or a continuous supervision of home-based exercises after the intervention program must be offered to prevent a decline in LSM.

### Sample characteristics

Only an insignificant number of studies (n = 4) had a male predominance. This is in line with a previous review observing a female predominance in intervention studies [71]. Therefore, no conclusions regarding an influence of gender in the effect of the interventions can be drawn. Previous research, however, suggests a relationship between gender and LSM in older adults with women having a more restricted LSM [72, 73]. Therefore, women may have more room for improvement and thus may be more likely to benefit from LSM interventions.

Looking at the different study populations, it is apparent that all groups benefited most from multidimensional interventions. Furthermore, the results of this review show a slight tendency that healthy and community-dwelling older adults are less responsive to the interventions than nursing home residents or older adults with severe mobility limitations. Nakagawa et al. [45], for instance, performed a simple physical exercise program combined with home training and compared non-frail and frail older people. Only the latter group could significantly enhance their LSM. One explanation why LSM did not improve in the healthy group might be a ceiling effect of the assessment tool (LSA). At baseline, the healthy older adults reached an average score of 74.4 ± 16.1 points, which was significantly higher than the average LSA score found in other studies. However, more likely, it indicates that physical performance was not the main mobility-limiting factor in the healthy group as it was in the frail group. Lifestyle interventions such as motivational training or knowledge-building aspects might have produced better results for the group without mobility impairments. All in all, the effect of interventions seems to be indirectly related to participants’ health status, degree of independence (e.g., institutionalized or community-dwelling), and mobility limitations.

### Assessment tools

Objective measurement methods (GPS and indoor wireless sensor network) for surveying the effectiveness of interventions to increase LSM are severely underrepresented (n = 2) [34, 41]. Still, both studies using objective methods resulted in an improvement in LSM. Questionnaires like the LSA have the advantage of covering mobility independence respectively need for assistance which might be relevant for frail people. However, due to the potential “recall bias” and social desirability bias when answering questionnaires, objective survey methods have a higher validity [74, 75] and should theoretically be applied more often [76]. It must be considered, though, that devices that enable objective LSM mobility assessments usually come with higher costs than simple self-reported questionnaires and often show lower acceptance rates (especially in cases where additional devices need to be used for study purposes other than the ones participants already carry for their personal use,e.g., their own smartphones), hence their sparse use in intervention studies so far. Technological advancements will inevitably lead to more frequent use of objective assessment tools which will then enable a more fine-grained analysis of LSM [77] and it will be interesting to see whether the results of intervention approaches in the future will be in line with the results presented here.

### External confounders

Some of the included studies [40, 47, 49, 50] adjusted for person-specific covariates like age, gender, BMI, race, and living situation. However, previous research could show that external factors like seasons and especially cold weather also have an important impact on life-space mobility which is even more pronounced in older adults [78]. Only a few studies included in this review such as Levasseur et al. [43] explicitly suspected a seasonal influence on the change of LSM in their participants. Some other studies [45, 55, 58] also highlighted the importance of considering seasonal and weather effects when analyzing LSM, however, none of them integrated these factors or other potential external confounders in their analyses.

### Limitations

This systematic scoping review is of rather narrative nature and provides a more qualitative than quantitative evaluation of intervention effects on LSM. Scarcity of intervention studies aimed at improving LSM currently does not allow meta-analytical approaches but given that this field of work’s current increasing relevance, more studies can be expected. This will allow future meta-analysis to make a more robust statement regarding which interventions yield the best results for the increase of LSM in older adults. Although PubMed and Web of Science are very exhaustive and popular databases, it could still be that some studies were missed.

## Conclusions

Interdisciplinary intervention approaches comprising physical exercise combined with counseling or educational interventions seem to have the highest potential for the improvement of LSM in older adults. As expected, individuals with poorer health and/or mobility limitations tend to profit more from such interventions compared to healthier individuals without mobility impairments. Considering that an increase in LSM is beneficial even for individuals without restricted LSM in order to build a reserve against a loss of mobility and the associated negative consequences, it remains interesting to see whether interventions targeting specifically high-functioning individuals would have additional advantages in their quality of life.

Naturally, the most effective interventions are the ones that are specifically designed to improve LSM. In addition, it has been shown that most effective interventions at least partly included physical performance training. However, according to the results of this review and to previous studies, physical factors are especially, but not solely associated with LSM, which is why counseling and educational interventions must also be regarded as an important part of multifactorial interventions for different target groups.

The current empirical evidence is still too small for profound recommendations concerning the most appropriate intervention strategies for the respective target groups, but future meta-analyses will be helpful to provide a quantitative evaluation of LSM enhancing interventions once possible.

## Electronic supplementary material

Below is the link to the electronic supplementary material.

Supplementary Material 1

Supplementary Material 2

Supplementary Material 3

Supplementary Material 4

## Data Availability

All data analyzed during this study are included in this published article [and its supplementary information files].

## Abbreviations

GPS: Global Positioning System
HBMA: Homebound Mobility Assessment
LSA: Life-Space Assessment
LSM: Life-Space Mobility
LSQ: Life-Space Questionnaire
NIH: National Institutes of Health
NHLSD: Nursing Home Life-Space Diameter
PRISMA-ScR: Preferred Reporting Items for Systematic reviews and Meta-Analyses extension for Scoping Reviews
RCT: Randomized Controlled Trial
