# Supplementary material for: Multidimensional interventions to increase life-space mobility in older adults ranging from nursing home residents to community-dwelling: a systematic scoping review
Source: BMC Geriatr. 2023 Jul 6;23:412. doi: 10.1186/s12877-023-04118-3 (PMC10327334; doi:10.1186/s12877-023-04118-3)
Supplement: Supplementary file 2 — Supplementary Material 2 [file 12877_2023_4118_MOESM2_ESM.docx]

**Supplementary Table 2.** Methods, Results and Quality Assessment of the Studies included in this Review

| **Study** | **Sample** | **Study**  **design** | **Intervention type and control group** | **Time frame** | **LSM^a^ assessment tool & outcome** | **Primary out-come^b^** | **Results (LSM)** | **NIH^c^ Quality Assess-ment** |
| --- | --- | --- | --- | --- | --- | --- | --- | --- |
| Brienza et al. (2018) [1] | 258 participants (**IG^d^**: n=127, **CG^e^**: n=131)  89.0±8.9 years,  78.3 % female, nursing home residents using wheelchairs and at risk for pressure injuries | RCT^f^ | **IG: new individually configured lightweight manual wheelchair** coached and assessed in basic wheelchair skills, skin protection cushion, appropriate armrests etc., and further adjustments if required  **CG: skin protection cushion and minimal adjustments** | **26-weeks intervention period**  Assessment points: before and after the intervention | **NHLSD^g^**  primary outcome | n.a^h^ | **IG:** at baseline 29.0±16.5, post-intervention 31.03±16.17 points **CG**: at baseline 29.09±14.71, post-intervention 26.66±15.38 points  **Mean within-group difference:**  **IG**: 2.03±16.95, **CG**: -2.44±15.63  **> no significant difference in mean score between the groups after the intervention** (p=0.07) **> no significant difference in mean change score between the groups** (p=0.07) | Fair |
| Brown  et al. (2016) [2] | 100 participants (each group n=50), 73.9 years,  3% female, hospitalized, different medical conditions (most commonly lung or heart problems) | RCT | **IG: mobilization program** (walking program)  **+ behavioral Intervention** (encouragement of activity, out-of-bed protocol, daily goals)  **CG:** visits of a research assistant to control for the daily attention, diary for documentation of visitors | **Mean intervention period:** **3.6±2.3 days (range 1-21),** 7 days/week, 2 sessions/day, each 15-20 minutes   Assessment points: baseline and 1 month after discharge from hospital | **LSA^i^**  primary outcome | n.a. | **IG:** at baseline 53.9±4.15, after 1 months 52.6±4.39 points **CG:** at baseline 51.5±2.99, after 1 months 41.8±3.15 points  **Mean within-group difference:  IG:** -1.3 points, **CG**: -9.7 points  **Mean between-group-difference (at 1-month posthospitalization)**:  10 points (p=0.02)  **> IG significantly greater life-space than CG 1 month after discharge from hospital and less decline** | Good |
| Collins  et al. (2018) [3] | 72 participants (each group: n=24), 74.2±6.6 years, 74% female, community-dwelling, mobility limitations, osteoarthritis, mean BMI within obese range (30.2±6.3) | RCT | **2 intervention groups:**  **IG 1: Timing and coordination of gait training:** training of stepping and walking patterns in small groups, complex tasks combining different activities  **IG 2: Outdoor aerobic walk training**  **CG: Stretching and relaxation training** | **12-week intervention program**,  2 sessions/week, each 60 minutes  Assessment points: baseline (T0), after 12 weeks (T1), and after 24 weeks (T2, follow-up) | **LSA**  secondary outcome | Energy cost of walking (mLO2/kg/m) (5-minute treadmill walking test) | **IG1:** at baseline 65.6, after 12 weeks 79.5, after 24 weeks 71.4 points  **IG2:** at baseline 63.1, after 12 weeks 63.6, after 24 weeks 67.6 points  **CG**: at baseline 53.1, after 12 weeks 64.3, after 24 weeks 64.9 points  **> no "meaningful improvement" in any IG**  **Mean between-group difference** (at T1 and T2):  **IG1 vs. CG:** 8.5 (p=0.204) and 0.7 points (p=0.989)  **IG2 vs. CG:** -4.9 (p=0.585) and -0.8 points (p=0.986)  **> no significant difference between IG1, respectively IG2 and CG after the intervention** | Good |
| Crist  et al. (2021) [4] | 305 participants (**IG:** n=150; **CG:** n=155),  83 years,  73% female,  retirement community-dwelling | RCT | **IG: walking intervention:** encourage neighborhood walking by group education sessions, individual counseling, pedometers + educational materials, step counts, walking maps, and community improvements (e.g. extending crosswalk times, clearing pedestrian paths of hazards, adding wheel paths)   **CG: healthy aging control condition:** similar level of attention via group meetings and counseling calls, focus on topics related to successful aging (e.g. nutrition, sleep, general health) | **1-year intervention period**  Assessment points: baseline, at month 3,6,9, and 12 (at each point measurement for 6 days) | **GPS^j^ and accelero-meter**  Outcomes: total time and time spent walking in four life-space domains (Home, Retirement Community Campus, Neighborhood, and beyond the Neighborhood)   primary outcome |  | **>IG increased total walking in Campus, neighborhood and beyond neighborhood compared to CG >intervention did not affect overall time spent in non-home domains**    >out of total time spent in each domain, IG spent a greater percentage of time walking all domains and time points compared to CG >most sustained change in the Campus domain **> indicates that the intervention was effective specifically in increasing PA in further life-space domains (but not in the time spent in life-spaces)** | Fair |
| Crotty  et al. (2019) [5] | 240 participants (**IG:** n=121, **CG:** n=119), **IG:** 88.6±5.4 years, 73.1%; **CG:** 88.6±5.7 years, 75.2% female;  nursing home residents, recovering from hip fracture surgery, cognitive limitations | RCT | **IG:** **multidisciplinary postoperative rehabilitation program** (conducted in nursing care facilities): Comprehensive Geriatrics Assessment, Physiotherapy (mobility and task specific training, muscle strengthening, training of care staff and family), nutritional assessment and care plan | **4-week intervention program** (starting  within 24 h of return to the nursing home),  13 hours of input  Assessment points: baseline, at 4 weeks and at 12 months | **NHLSD**  primary  outcome | n.a. | **IG:** at baseline 0, after 4 weeks 8.2±0.47, after 12 months 10.5±0.63 points **CG:** at baseline 0 points, after 4 weeks 6.3±0.5, after 12 months 10.1±0.6 points **Mean between-group difference**: **after 4 weeks**: -1.9 points (95% CI^q^: -3.3 -0.57; p=0.0055);  **after 12 months**: 0.37 points (95% CI: -2.1- 1.3; p=0.6777)  **> IG significantly greater life-space than CG after intervention, but improvements were not sustained at 12 months** | Good |
| Fairhall et al. (2012) [6] | 241 participants (**IG**: n=120; **CG**: n=121), 83.3±5.9 years,  68% female,  community-dwelling, frail | RCT | **IG: multifactorial, interdisciplinary intervention**: supervised and home-based physiotherapy targeting mobility and physical activity, to prevent falls; interdisciplinary intervention based on present frailty criteria (e.g., occupational therapy, community exercise programs, dietary etc.)  **CG: usual care** | **12-months intervention program,** 10 physiotherapy sessions, each 45-60 minutes, 3-5 times/week independently but reviewed home exercise program  Assessment points: baseline, at 3 and at 12 months | **LSA**  primary  outcome | n.a. | **IG:** at baseline 27.6±12.9, at 3 months 35.5±16.1, at 12 months 34.2±16.2 points;  **CG:** at baseline 30.0±14.3, at 3 months 30.3±13.9, at 12 months 30.9±5.5 points;  **Mean between-group difference:**  **at 3 months:** 6.57 points (95% CI: 3.6-9.5; p <0.001)  **at 12 months:** 4.68 points (95% CI: 1.4-7.9 p= 0.005)  **˃IG significantly greater life-space than CG at 3 and 12 months** | Good |
| Hewitt  et al. (2018) [7] | 221 participants (**IG:** n=113, **CG:** n=108), 86±7 years, 65% female, nursing home residents | RCT | **IG:  Stage 1: Individual Progressive Resistance Training + Balance Exercise:** in group setting (n≤10), HUR Health and Fitness Equipment  **Stage 2: maintenance program:** resistance, weight bearing balance and functional group exercise sessions,  *dosage not progressed!* **CG: Usual care** | **Stage 1**: **25-week intervention program,** 2 sessions/week, each 60 minutes  **Stage 2: 6-months** maintenance program, 2 sessions/week, each 30 minutes  Assessment points: baseline, at 6 months, at 12 months | **LSA**  secondary  outcome | Rate of falls | **IG:** at baseline 34.56±18.56, at 6 months 44.07±19.81, at 12 months 41.72±22.37 points **CG:** at baseline 30.06±15.94, at 6 months 39.51±20.06, at 12 months 36.91±21.18 points  **> no significantly different improvement between IG vs. CG (p=0.667, effect size: 0.22)** | Good |
| Hiyama et al. (2019) [8] | 104 participants (**IG:** n=36;  **CG**: n=68),  **IG**: 69.7±6.1 years, 86.1% female; **CG:** 71±5.8 years, 80.9% female; underwent total knee arthroplasty within the past 3-6 months;  **CG**: patients not participating in the intervention due to time problems | Non-rando-mized  con-trolled trial | **IG: Walking event/walking tasks:** warming up with some flexibility and strength exercises, 3.5-km walking course with tasks like crossing a crosswalk, walking up- and downstairs without a railing, walking along dirt roads, and walking up and down a slope.   **CG: no intervention** | **1 walking event for 90 minutes**  Assessment points: 1 week before and at 1 month after the intervention | **LSA**  primary  outcome | n.a. | **IG:** at baseline 80.5±19.6, after 1 month 96.3±17.5 points **CG:** at baseline 81.2±21.5, after 1 months 81.1±21.3 points  **Mean between-group difference post-intervention:**  13.9 points (95% CI: 12.4-15.5, p<0.001)  **>IG significantly greater life-space than CG 1 month post intervention** | Fair |
| Jansen  et al. (2018) [9] | 143 participants (**IG:** n=78; **CG:** n=65), nursing home residents, severely impaired | Natural control  group/  Non-rando-mized controlled trial | **IG:** **PA-related intervention with several components**: (1) supervised group sessions, individual exercise, a serious game approach (dual motor/cognitive tasks), and specific training in severely impaired persons with focus on functional and strength exercises to improve key motor functions for mobility, increasing training intensity; (2) competence training of staff members, focus on PA-motivation and -engagement, implementation of training strategies in daily NH routine **CG: Natural control group: no intervention** | Training of the **nursing home residents:  12- week intervention** **program**, 2 sessions/week, each 45 minutes  Training of the **nursing home staff:  12- week intervention program**, 1 session/week, 8 1-hour sessions, 4 30-minutes sessions  Assessment points: baseline, after week 12-, and 3-months follow-up | **“Indoor wireless sensor network”** to assess life-space  primary outcome |  | **Between baseline and week 12: PA-IG^k^**: LSM-parameters increased   **Nat-CG^l^:** decline of Life-space score (LSSc) and the Maximal distal zone (MaxZ)   **> PA-IG significantly higher Life-space score** (LSSc) (β= 0.13, p=0.003), **spent more time away from private room** (TAFR) (β= 0.28, p=0.015), **and had a more extensive life-space** (MaxZ) (β=0 .29; p=0.003) **compared to Nat-CG**  **After 3 months**: **> Sustainability of effects after follow-up period only for MaxZ** (β=0.39, p=0.012) **and marginally for LSSc** (β=0.11, p=0.065) | Fair |
| Jensen  et al. (2004) [10] | 18 participants,  64.1±4.8 years, 100% female, BMI ≥ 30, community-dwelling, obesity comorbidities | Pre-post  inter-vention study | **weight loss program**: promoting prudent diet, behavior modification and physical activity | **3- month intervention program,** 8 sessions, each 30 minutes  Assessment points: baseline and after 3 months | **LSA**  secondary outcome | Health, function, and Quality of life | **> no significant improvement after 3 months (p=1.0)** | Fair |
| Kamga  et al. (2017) [11] | 80 participants (**IG:** n=41; **CG:** n=39), 76±12 years, 38% male,  age related eye disease, at least mild depressive symptoms | RCT | **IG: self-care tools plus coaching:** participants received a notebook with written tools and audio tools teaching skills such as reactivating of life, and problem solving and a Mood Monitoring tool, individual recommendations of additional tools (e.g., relaxation CD, emotional eating and information for family members); three 10-minute motivational phone calls  **CG: usual care** (after the study they received the notebook as well) | **8-weeks intervention program**   Assessment points: baseline, after 8 weeks | **LSA**  secondary outcome | De-pressive symp-toms (Patient Health  Ques-tionnaire-9) | **IG:** at baseline 46.3±28.1 points, at follow-up 44.1±22.2 **CG:** at baseline 45.0±23.7 points, at follow up 41.7±19.9  **Mean within-group difference:**  **IG**: -2.2±24.3 points **CG**: -3.9±19.6 points  **> no significant difference in mean change scores between IG and CG (p=0.715)** | Good |
| Kami-oka et al. (2020) [12] | 47 participants 76.7±11.1 years,  42.6% female,  users of home-based rehabilitation, most frequent disorders: 29.8% cerebrovascular, 23.4% osteoarticular | Pre-post study without control group | **Home-based rehabilitation program** physical exercises (range-of-motion, muscle-strengthening, relaxation), exercises and instructions relating to activities and participation (e.g. walking, transfer, going out)  **> individual interventions, no standardized plan** | **6- months intervention period**  different intervention frequencies:  highest frequency: 4 to <8 sessions/moth (59.6%),  each session typically 40 (61,76%) or 60 (34.0%) minutes  Assessment points: baseline, at 3 and 6 months | **LSA**  primary outcome | n.a. | At baseline: 25.2±18.0 points, after 3 months 27.8±16.5, after 6 months 30.2±19.7 points  **> significant difference among the three time points (p=0.025)**  **> significant improvement between baseline and 6 months (p=0.013)   >significant change of score in life-space level 2 (outside home) (p=0.004) and level 3 (neighborhood) (p=0.042)** | Fair |
| Kato et al. (2022) | 3 participants, 82.5±5.0 years, 66% female,  nursing home residents, severe care needs | Pre-post intervention study without control,feasi-bility study | **Implementation of a rise-assisting robot (Resyone):** used to facilitate visits of additional sites in and around the nursing home (as additional care operations) | **4-week intervention** | Places visited after the transfer (as indicated by caregivers)  Primary outcome | Other outcome: facial ex-pression | **>increase in frequency of visits to areas which had been visited infrequently or not at all before the intervention** (e.g., ID1: increase in frequency of visiting hall from 2% to 25%) | Fair |
| Le-vasseur et al. (2019) [13] | 16 participants,  76.4±7.6 years,  62.5% female,  community-dwelling, with (n=6) and without disability (n=10) | Pre-post study without control group | **Adapted French Version of the  "Lifestyle Redesign" (occupational therapy intervention):** empowers older adults to regularly perform healthy and fulfilling activities; group sessions (based on 12 modules, e.g. occupation, health, transportation) and individual meetings (help to engage in personalized meaningful activities) | **6-months intervention period,** 2 hour/week group sessions, 1 hour/month individual meeting  Assessment points: baseline (T1), post-intervention (T2), 3 months follow-up (T3), and 6 months follow-up (T4) | **LSA**  primary outcome (among others) | n.a. | **Results of the whole sample (n=16)** T1: 75.0±32.2 points,  T2: 63.0±24.5 points,  T3: 74.0±27.0 points,  T4: 77.0±27.6 points **> LSM significantly decreased between T1 and T2 and significantly increased between T2 and T4**  **> LSM significantly increased between T2 and T4 > no significant difference over the whole period (p=0.08)**   **Results of participants without disability (n=10)** T2 (73.0±8.3 points) vs. T1 (83.0±7.3 points): p=0.01  T2 (73.0±8.3 points) vs. T4 (82.0±6.3 points): p=0.02  **> LSM significantly decreased and increased again in participants without disability** | Good |
| Liddle  et al. (2014) [14] | 131 participants (**IG:** n=67; **CG:** n=64),  78.9±7.6 years,  74.1 % female, (planned) cessation of driving | RCT | **IG: UQDRIVE^m^ program - group education and support program:** 8-15 group members, information sharing, group activities and discussions, practical exercises, and outings (drawn from 7 modules, varied according to the needs of group members)  **CG: waitlist control group** | **6-months intervention period,** 6 intervention sessions,  1 session/week, each 3-4 hours  Assessment points: at baseline (2 weeks before the intervention) (Time 1), immediately after the intervention (Time 2), and at 3 months follow-up (Time 3) | **Question:** episodes leaving home per week  Primary outcome | n.a. | **Time 2:** **IG**: 5.9 episodes away from home,  **CG**: 4.4 episodes away from home  **> Significant effect of Intervention x Time 2** (ß=1.36, z=2.56, p=0.01, 95% CI: 0.32-2.40)  **> The effect was not maintained till Time 3** (Intervention x Time 3: ß=-0.54, z=-0.95, p=0.344, 95% CI: -1.66- 0.58) | Fair |
| Liu et al. (2021) [15] | 194 participants (**IG** n=93; **CG** n=101), 75.8±7.5 years, 84.6% female, low income, restricted daily activities | RCT | **IG: therapy sessions with an occupational therapist (OT) and a registered nurse (RN), and repairs modifications by a handyman:** six goals (three with OT and three with RN); OT goals: e.g., evaluating home safety, identifying behavioral and environmental contributors to performance difficulties; RN goals: identify how and whether pain, depression, strength, balance, medication management, and the ability to communicate with care practitioners impact daily function; structural adaptions and home repairs by the handyman  **CG: attention-control group**: identified sedentary activities they wanted to do | **4-months intervention period** 6 OT sessions, each 1 hour;  4 RN sessions, each 1 hour; 2 sessions with the handyman: one 1-hour visit and one full day's work;    Assessment points: baseline and at 5 months | **HBMA^n^**   primary outcome | n.a. | **Life-space change at 5 months:**  **IG**: decreased: 27.1%, unchanged 24.7%, improved 48.2%; **CG**: decreased 44.7%, unchanged 16.0%, improved 39.3%  **> More IG participants improved their LSM  > IG participants were more likely to have improved vs. decreased life-space in the areas of stairs** (adjusted OR^r^ = 4.09; 95% CI = 1.34–12.48, p<0.05), **leaving the house other than for health care** (adjusted OR = 2.40; 95% CI = 1.01–5.73, p<0.05) **and overall life-space** (adjusted OR = 2.15; 95% CI = 1.10–4.19, p<0.05) | Low |
| Maki-zako  et al. (2019) [16] | 89 participants (**EG:** n=30; **HG:** n=30; **CG** n=29),  73.1±5.5. years,  50.6% female,  depressive symptoms, memory problems | RCT | 3 groups:  **1. Exercise group (EG)**: **multicomponent exercise program**: aerobic exercises, muscle strength training, postural balance retraining, and dual-task training (group training with 9-12 individuals/class) **2. Horticulture group (HG)**: **horticultural activity program**: nature-based group activities like crop-related activities (cultivating, growing, and harvesting), gardening activities like group planting, nutritional information, and recipes from vegetable experts **3. Control group (CG)**: **Educational control group:** topics considered less likely to influence study outcomes (e.g., traffic safety and disaster prevention) | **6-months intervention period,**  **EG and HG**: 20 sessions,  1 session/week, each 90 minutes  **CG**: Two 90-minute classes  Assessment points: baseline, after 6 months and at 12-month follow-up | **LSA**   other outcome | De-pressive symp-toms  and memory | **EG:** at baseline 80.9±16.7 points, after 6 months 80.4±12.2, after 12 months 82.2±18.2; **HG**: at baseline 86.6±17.8 points, after 6 months 83.3±19.2, after 12 months 78.3±24.3 **CG**: at baseline 90.4±16.9 points, after 6 months 78.9±16.1, after 12 months 81.7±27.6  M**ean within-group difference** (baseline vs. 6 post-intervention and baseline vs. 12 months follow-up) **EG**: -0.5±19.2 (p>0.05), 2.7±19.7 points (p>0.05) **HG**: -4.4±15.6 (p>0.05), -9.6±20.6 points (p>0.05) **CG**: -12.0±19.5, -14.4±29.7 points (p>0.05)  **> no significant within-group differences/significant change in either group > no significant between-group differences between pre-intervention and 12 month follow-up** | Good |
| Matsuda et al. (2015) [17] | 42 participants  76±3.4 years,  100% female,  potentially requiring nursing care or support | pre-post  inter-vention study without control gorup | **Physical training:** stretching, muscle strength training, balance training, instructions in walking, and individualized instruction; group training for stretching and balance exercises | **3-months intervention period,** 2 sessions/week   Assessment points: before and after the intervention (not closer specified) | **LSA**  primary outcome (among others) | Other: strength, flexi-  bility,  balance, gait, trunk stability, Falls efficacy | **Pre- and post-intervention:**  49.5±14.5 and 53.5±15.9 points (p<0.05)  **> LSA score increased significantly** | Fair |
| Mura-bayashi et al. (2019) [18] | 115 participants (**MT:** n=58, 80.9±5.6 years; **CG** n=57, 81.7±5.5. years), 93.6% female, frail, in need of care, needs regarding social withdrawal, dementia, or depression | RCT  (Con-trolled cross-over Study) | **Music Therapy group (MT):** musical greeting and physical exercise with music, singing, rehabilitative activities, listening   **CG: waiting control group** | **12-weeks intervention period** (first period), 1 session/week, each 45-50 minutes  4-week washout period  **12 weeks in alternate group** (second period)  Assessment points: baseline 1, after 12 weeks, after 16 weeks (baseline 2) and after 28 weeks (4 weeks washout period) | **LSA**   primary outcome (among others) | Other out-comes:  cognitive and physical function, psycho-physical health, IADL (…) | **Mean within-group difference** (from baseline (first and second period)):  **MT**: -2.58 points and 0.55 points **CG**.: -3.72 points and 0.69 points  **> no significant treatment effect (p=0.83), treatment-period interaction (p=0.88), or period effect (p=0.21)** | Good |
| Naka-gawa  et al. (2008) [19] | 74 participants **“General elderly”**: (n=44, 74.2±4.38 years, 38 women) community-dwelling;  **Frail elderly**: (n=30, 80.6±6.12 years, 20 women),  in need of support or care, using day rehabilitation service one or two days a week | pre-post inter-vention  study with two intervention groups | **Both groups:  simple exercise program:**  **main exercises:** 8 muscle training exercises for major muscles in the lower legs, trunk, 5 stretching exercises (always carried out) **combined exercises**: postural and light exercises (flexibly carried out)  **individual exercises for home trainin**g (3-5 of the exercises above) | **No unified period of observation and frequency of sessions**  **Average intervention period: General elderly:**  81.4±3 days, 1 session every 2 weeks, each 20 minutes **Frail elderly group:**  90.2±2.9 days, 1-2 sessions/week (provided 5 days/week), each 20 minutes  Assessment points: at baseline and after 3 months | **LSA**   primary outcome (among others) | Other out-comes:  body weight, strength, TUG, functional reach test (…) | **General elderly group:**  at baseline: 74.4±16.1 points, after the intervention: 78.1±18.4 points  **> no significant improvement (p=0.077)  Frail elderly group:** at baseline: 35.4±13.0 points, after the intervention: 39.6±15.2 points **>significant improvement (p=0.0004)** | Good |
| Siltanen et al. (2020) [21] | 204 participants (**IG**: n=101; **CG**: n=103), **IG**: 74% 75 years, 26% 80 years, 60% female, **CG**: 75% 75 years, 25% 80 years, 61% female;  community-dwelling | RCT | **IG: counseling aimed at increasing self-selected, primarily out-of-home activity:** face-to-face counseling session and phone calls + supportive materials (e.g., active aging information, calendar, newsletter with information on activities available and success stories)  **CG: general health information** (exercise, nutrition, cardiovascular diseases, and type 2 diabetes) | **12-months intervention program,**  1 90-minutes face-to-face counseling,  4 shorter phone counseling sessions at months 1,3,6,9   Assessment points:  baseline and at 12-month follow-up | **LSA**  secondary outcome | **Active Aging total score**  (Uni-versity of Jyväs-kylä  Active Aging Scale (UJACAS)) | **IG**: at baseline 74.4±9.2 and at 12 months 76.3±14.5 points **CG**: at baseline 74.7±9.3 and at 12 months 74.9±13.6 points  **> no significant time effect (p=0.807) > no significant group effect (p=0.482) > no significant group-by-time effect (p=0.409)** | Good |
| Swan  et al. (2019) [22] | 40 participants (each group n=10),  72±9.8 years, 30% female,  treated respiratory disease (Medical Research Council breathlessness scale grade ≧3) | Feasi-bility study/ phase II RCT | 4 groups: **1. Handheld fan + exercise advice  2. Calming hand (CH) + exercise advice 3. Fan + CH + exercise advice 4. Exercise advice alone  Exercise training + information leaflet for the use at home**: standardized breathlessness self-management and exercise advice **Handheld fan and calming hand (CH) + information leaflet**: participants are instructed to use it/them whenever they feel breathless | **28-days intervention period**  One hour face-to-face individual exercise training  One further training for the participants allocated to the CH and/or fan groups  Assessment points: baseline and at day 28 | **LSQ^o^**  primary outcome (among others) | Other outcomes e.g.:  accepta-bility, data quality, recovery time from  exertion,  General self-efficacy | **Exercise advice**: at baseline 54.45±24.74 points, at day 28 54±24.21  > **mean change -0.45 points**  **Fan & exercise advice**: at baseline: 60.8±22.69 points, at day 28: 64.05±19.23  > **mean change 3.2 points**  **CH & exercise advic**e: at baseline: 47.05±13.9 points, at day 28: 55.2±14.67  > **mean change 8.15 points**  **Fan & CH & exercise advice**: 55.5±19.37 points, at day 28: 61.05±27.06  **> mean change 5.55 points** | Fair |
| Tay et al.  (2022)  [23] | 81 participants (**IG**: n=52; **CG** : n=29),  **IG** : 69.8±6.2 years and 76.9% female,  **CG:** 69.8±7.0 years and 82.8% female;  Prefrail, community-dwelling | Non-randomized | **IG: multi-disciplinary intervention program:**  individual counseling session, group exercise class (strength, balance and endurance training), home exercises, and nutritional intervention (didactics, food-based games and grocery-shopping trips)  **CG (participants declining enrolment in IG):** individual **counseling session**, 4 group-based education classes on frailty | **4-months intervention period**  1 counseling session  1 session/week exercise program, each 60 minutes  3 sessions/week home exercises  1-2 sessions/month nutritional intervention, each 90 minutes  Assessment points: baseline and 1-year follow up | **LSA**  Secondary outcome | Frailty, intrinsic capacity | **IG**: at baseline 77.6±25.0 points, after 1 year 75.7±21.8  **CG**: at baseline 85.5±17.7 points, after 1 year 82.9±16.1  **Mean within group difference:**  **IG:** -1.82±20.2 points  **CG:** -2.2±16.1 points  **>no significant within-group difference**  **>no significant between-group difference after 1 year** (p=0.124)  **> no significant between group difference in mean change score** (p=0.933) | Low |
| Tanaka et al. (2021) [24] | 31 participants (**IG:** n=16; **CG:** n=15),  **IG**: 88.1±8.1 years and 62.5% female,  **CG**: 84.2±74. years and 53.3% female; older people with dementia in a geriatric health service facility | Quasi-rando-mized con-trolled trial | **IG: group-based activity (conducted by a physical therapist and an occupational therapist):** reality orientation, seated exercises (stretching, muscle strength training, aerobic exercise), cognitive training or stimulation (e.g., music-based activity, reminiscence, facial expression, stimulation of verbal fluency), cool down  **CG: usual care** (=rehabilitation for 20 min, 3 times/week) | **8-weeks intervention program**, 2 sessions/week, each 45 minutes  Assessment points: baseline and after 8 weeks | **NHLSD**  secondary  outcome | Quality of Life, Nurses' observa-tion Scale of Geriatric Patients (NOSGER) | **IG**: at baseline 48.2±15.7 points, after 8 weeks 48.4±14.8  **CG:** at baseline 41.0±11.8 points, after 8 weeks 45.0±10.6   **Mean within-group difference:  IG**: 0.2±1.7 points (p=0.919) **CG**: 2.6±4.1 points (p=0.551)  **Mean between-group difference:**  -2.4 points (95% CI: -10.4-5.5)  **> no significant improvement in IG or CG > no significant difference between IG and CG** | Fair |
| Todo  et al. (2021) [25] | 30 participants,  82.4±7.5 years,  80% female,  patients of a home-based rehabilitation service facility, restricted life-space (LSA<52.3) | Pre-post  Inter-vention  study | **Multicomponent home-based rehabilitation** **program:** (conducted by physical and occupational therapists): exercises (stretching, muscle training, balance), practicing ADL (transfer, gait, fall-recovery techniques etc.), ADL techniques (eating, toileting, dressing etc.), instrumental ADL techniques (e.g. meal preparation, laundry), improving home environment, and caregiver support (safer techniques for assisting etc.) | **3-months intervention period**   Assessment points: baseline, after 3 months | **LSA**  primary  outcome | n.a. | Median at baseline 12.0 points (IQR^s^: 16.8), after 3 months 30.5 points (IQR 15.3)  **>significant improvement** (p<0.001)  Proportion of participants at maximal life-space level 5 (unlimited mobility): at baseline 16.7%, after 3 months 33.3% | Good |
| Uemura et al. (2021) [26] | 60 participants (each group n=30), 74.0±4.9 years, 66.7% female,  community-dwelling, low health literacy | RCT | **IG: active learning program:** exploratory learning,  group work, and self-planning of behavior changes that promote a healthy lifestyle **CG: didactic teaching method Both groups:** Focus on role of exercise, diet, and cognitive activity for promoting health among older adults | **24-week intervention program,** 1 session/week, each 90 minutes  Assessment points: baseline, week 24 | **LSA**  secondary outcome | Comprehensive Health  Literacy  (Health Literacy Scale (HLS)-14) | **Mean within-group difference:**  **IG**: 14.2 points (95% CI: 5.7-22.6),  **CG**: 1.3 points (95% CI: -7.0-9.7)  **Between group difference in mean change score:**  12.8 points (95% CI: 0.9-24.7, p=0.035)  **>Significant improvement in IG compared to CG** | Good |
| Ullrich  et al. (2021) [27] | 118 participants (**IG:** n=63, **CG:** n=55),  **IG**: 82.2±5.8 years and 76.2% female,  **CG**: 82.4±6.2 years and 76.4% female  MMSE: 23.3±2.4/ cognitive impairment (CI), physical impairment, recently discharged from geriatric rehabilitation (reasons for hospital admission: cardiovascular (20%), degenerative joint (18%), neurological (16%) diseases and consequences of falls (14%) | RCT | **IG: CI-specific, home-based strength, balance and walking training + tailored motivational strategies:** participants were instructed to independently perform the training and walking course as a daily routine, large poster with exercises and a printed manual were handed out, CI-specific motivational approach included social support, goal-setting, identification and removal of barriers to exercise and walking and self-monitoring via training diary and pedometer   **CG: placebo activity:** received a training manual with unspecific flexibility and strength exercises and a newsletter-based information on nutrition and relaxation | **12- week intervention period**  Both groups: 5 home visits with decreasing frequency, phone calls once/week; no support/contact during follow-up period    Assessment points: before randomization (T1), after 12-weeks (T2) and after 12-week follow-up period (T3) | **LSA – CI^p^**  primary outcome (but in a secondary analysis) |  | **>LSA-CI composite score:** Significant mean group difference at T2 (when controlled for T1 value and gender) (8.15 points, 95% CI: 2.89-13.41, p=0.003)  **> Independent life-space sub-score:** Significant group difference at T2 (when controlled for T1 value and gender) (0.39 points, 95% CI: 0.00-0.78, p=0.048)  **> no significant difference between IG and CG regarding other sub-scores** (LSA-CI-M: 0.33 points, 95% CI: -0.18-0.84, p=0.208; LSA-CI-E: 0.43 points, 95% CI: -0.11-0.98, p=0.122)  **> Training gains decreased after the intervention with no significant group difference at T3** (LSA-CI-C: 3.03 points, 95% CI: -2.39-8.46, p=0.274) | Good |

*Note*. ^a^ LSM: Life-Space Mobility; ^b^ Primary outcome specified in case that LSM is not the primary outcome; ^c^ NIH: National Institutes of Health; ^d^ IG: Intervention Group; ^e^ CG: Control Group; ^f^ RCT: Randomized Controlled Trial; ^g^ NHLSD: Nursing Home Life-Space Diameter; ^h^ n.a.: not applicable; ^i^ LSA: Life-Space Assessment; ^j^ GPS: Global Positioning System; ^k^ PA-IG: Physical Activity-Intervention Group; ^l^ Nat-CG: Natural Control Group; ^m^ UQDRIVE: University of Queensland Driver Retirement Initiative; ^n^ HBMA: Homebound Mobility Assessment;

^o^ LSQ: Life-Space Questionnaire; ^p^ LSA-CI: Life-Space Assessment in Persons with Cognitive Impairment, ^q^CI: Confidence Interval; ^r^OR: Odds Ratio; ^s^IQR: Interquartile Range

**References**

1. Brienza DM, Karg PE, Bertolet M, Schmeler M, Poojary-Mazzotta P, Vlachos H, et al. A Randomized Clinical Trial of Wheeled Mobility for Pressure Injury Prevention and Better Function. J Am Geriatr Soc. 2018;66:1752–9.

2. Brown CJ, Foley KT, Lowman JDJ, MacLennan PA, Razjouyan J, Najafi B, et al. Comparison of Posthospitalization Function and Community Mobility in Hospital Mobility Program and Usual Care Patients: A Randomized Clinical Trial. JAMA Intern Med. 2016;176:921–7.

3. Collins KJ, Schrack JA, VanSwearingen JM, Glynn NW, Pospisil MC, Gant VE, et al. Randomized Controlled Trial of Exercise to Improve Walking Energetics in Older Adults. Innov aging. 2018;2:1–10.

4. Crist K, Jankowska MM, Schipperijn J, Rosenberg DE, Takemoto M, Zlatar ZZ, et al. Change in GPS-assessed walking locations following a cluster-randomized controlled physical activity trial in older adults , results from the MIPARC trial. Heal Place. 2021;69 April:102573.

5. Crotty M, Killington M, Liu E, Cameron ID, Kurrle S, Kaambwa B, et al. Should we provide outreach rehabilitation to very old people living in Nursing Care Facilities after a hip fracture? A randomised controlled trial. Age Ageing. 2019;48:373–80.

6. Fairhall N, Sherrington C, Kurrle SE, Lord SR, Lockwood K, Cameron ID. Effect of a multifactorial interdisciplinary intervention on mobility-related disability in frail older people: randomised controlled trial. BMC Med. 2012;10.

7. Hewitt J, Goodall S, Clemson L, Henwood T, Refshauge K. Progressive Resistance and Balance Training for Falls Prevention in Long-Term Residential Aged Care: A Cluster Randomized Trial of the Sunbeam Program. J Am Med Dir Assoc. 2018;19:361–9.

8. Hiyama Y, Kamitani T, Mori K. Effects of an Intervention to Improve Life-Space Mobility and Self-Efficacy in Patients following Total Knee Arthroplasty. J Knee Surg. 2019;32:966–71.

9. Jansen CP, Diegelmann M, Schilling OK, Werner C, Schnabel EL, Wahl HW, et al. Pushing the Boundaries: A Physical Activity Intervention Extends Sensor-Assessed Life-Space in Nursing Home Residents. Gerontologist. 2018;58:979–88.

10. Jensen GL, Roy M-A, Buchanan AE, Berg MB. Weight loss intervention for obese older women: improvements in performance and function. Obes Res. 2004;12:1814–20.

11. Kamga H, McCusker J, Yaffe M, Sewitch M, Sussman T, Strumpf E, et al. Self-care tools to treat depressive symptoms in patients with age-related eye disease: a randomized controlled clinical trial. Clin Experiment Ophthalmol. 2017;45:371–8.

12. Kamioka Y, Miura Y, Matsuda T, Iijima Y, Suzuki A, Nakazato K, et al. Changes in social participation and life-space mobility in newly enrolled home-based rehabilitation users over 6 months. J Phys Ther Sci. 2020;32:375–84.

13. Levasseur M, Filiatrault J, Larivière N, Trépanier J, Lévesque M-H, Beaudry M, et al. Influence of Lifestyle Redesign(®) on Health, Social Participation, Leisure, and Mobility of Older French-Canadians. Am J Occup Ther Off Publ Am Occup Ther Assoc. 2019;73:7305205030p1–18.

14. Liddle J, Haynes M, Pachana NA, Mitchell G, McKenna K, Gustafsson L. Effect of a Group Intervention to Promote Older Adults’ Adjustment to Driving Cessation on Community Mobility: A Randomized Controlled Trial. Gerontologist. 2014;54:409–22.

15. Liu M, Xue Q-LL, Gitlin LN, Wolff JL, Guralnik J, Leff B, et al. Disability Prevention Program Improves Life-Space and Falls Efficacy: A Randomized Controlled Trial. J Am Geriatr Soc. 2021;69:85–90.

16. Makizako H, Tsutsumimoto K, Doi T, Makino K, Nakakubo S, Liu-Ambrose T, et al. Exercise and Horticultural Programs for Older Adults with Depressive Symptoms and Memory Problems: A Randomized Controlled Trial. J Clin Med. 2019;9.

17. Matsuda K, Ikeda S, Nakahara M, Ikeda T, Okamoto R, Kurosawa K, et al. Factors affecting the coefficient of variation of stride time of the elderly without falling history: a prospective study. J Phys Ther Sci. 2015;27:1087–90.

18. Murabayashi N, Akahoshi T, Ishimine R, Saji N, Takeda C, Nakayama H, et al. Effects of Music Therapy in Frail Elderlies: Controlled Crossover Study. Dement Geriatr Cogn Dis Extra. 2019;9:87–99.

19. Nakagawa K, Inomata N, Konno Y, Nakazawa R, Hagiwara K, Sakamoto M. The Characteristic of a Simple Exercise Program under the Instruction of Physiotherapists-For General Elderly People and Frail Elderly People. J Phys Ther Sci. 2008;20:197–203.

20. Ross LA, Schmidt EL, Ball K. Interventions to maintain mobility: What works? Accid Anal Prev. 2013;61:167–96.

21. Siltanen S, Portegijs E, Pynnönen K, Hassandra M, Rantalainen T, Karavirta L, et al. Effects of an Individualized Active Aging Counseling Intervention on Mobility and Physical Activity: Secondary Analyses of a Randomized Controlled Trial. J Aging Health. 2020;32:1316–24.

22. Swan F, English A, Allgar V, Hart SP, Johnson MJ. The Hand-Held Fan and the Calming Hand for People With Chronic Breathlessness: A Feasibility Trial. J Pain Symptom Manage. 2019;57:1051-1061.e1.

23. Tay L, Tay EL, Mah SM, Latib A, Ng YS. Intrinsic capacity rather than intervention exposure influences reversal to robustness among prefrail community-dwelling older adults: A non-randomized controlled study of a multidomain exercise and nutrition intervention. Front Med. 2022;9.

24. Tanaka S, Yamagami T, Yamaguchi H. Effects of a group-based physical and cognitive intervention on social activity and quality of life for elderly people with dementia in a geriatric health service facility: a quasi-randomised controlled trial. Psychogeriatrics. 2021;21:71–9.

25. Todo E, Higuchi Y, Ueda T, Murakami T, Kozuki W. A 3-month multicomponent home-based rehabilitation program for older people with restricted life-space mobility: a pilot study. J Phys Ther Sci. 2021;33:158–63.

26. Uemura K, Yamada M, Okamoto H. The Effectiveness of an Active Learning Program in Promoting a Healthy Lifestyle among Older Adults with Low Health Literacy: A Randomized Controlled Trial. Gerontology. 2021;67:25–35.

27. Ullrich P, Werner C, Bongartz M, Eckert T, Abel B, Schönstein A, et al. Increasing Life-Space Mobility in community-dwelling older persons with cognitive impairment following rehabilitation: A randomized controlled trial. J Gerontol A Biol Sci Med Sci. 2020. https://doi.org/10.1093/gerona/glaa254.
