# Supplementary material for: Multidimensional interventions to increase life-space mobility in older adults ranging from nursing home residents to community-dwelling: a systematic scoping review
Source: BMC Geriatr. 2023 Jul 6;23:412. doi: 10.1186/s12877-023-04118-3 (PMC10327334; doi:10.1186/s12877-023-04118-3)
Supplement: Supplementary file 3 — Supplementary Material 3 [file 12877_2023_4118_MOESM3_ESM.docx]

**Supplementary Table 3.** Quality Assessment for before-after (pre-post) studies with no control group (NIH Quality Assessment Scale)

| **Author** | **1^a^** | **2^b^** | **3^c^** | **4^d^** | **5^e^** | **6^f^** | **7^g^** | **8^h^** | **9^i^** | **10^j^** | **11^k^** | **12^l^** | **points** | **Quality** |
| --- | --- | --- | --- | --- | --- | --- | --- | --- | --- | --- | --- | --- | --- | --- |
| Jensen et al. (2004) [1] | yes | yes | yes | yes | no | yes | yes | NR^m^ | no | yes | no | NA^n^ | 7 | Fair |
| Kamioka et al. (2020) [2] | yes/no | yes | yes | yes | NR | no | yes | no | no | yes | yes | NA | 6,5 | Fair |
| Kato(2022) [3] | yes | yes | yes | NR | no | yes | no | no | yes | no | no | NA | 5 | Fair |
| Levasseur et al. (2019) [4] | yes | yes | yes | NR | yes | no | yes | NR | yes | yes | yes | NA | 8 | Good |
| Matsuda et al. (2015) [5] | yes | no | NR | yes | no | yes | yes | NR | NR | yes | no | NA | 5 | Fair |
| Nakagawa et al. (2008) [6] | yes | yes | yes | yes | NR | yes | yes | no | yes | yes | no | NA | 8 | Good |
| Todo et al. (2021) [7] | yes | yes | yes | yes | no | yes | yes | no | yes | yes | no | NA | 8 | Good |

Note. ^a^ 1: Study objective clearly stated; ^b^ 2: eligibility criteria prespecified and clearly described; ^c^ 3: participants representative; ^d^ 4: all eligible participants enrolled; ^e^ 5: sample size sufficiently large; ^f^ 6: intervention clearly described and delivered consistently; ^g^ 7: outcomes prespecified, valid, reliable and assessed consistently; ^h^ 8: assessors blinded; ^i^ 9: loss to follow-up ≤ 20% and those lost accounted for in the analysis; ^j^ 10: statistical methods examine changes, p-values provided; ^k^ 11: interrupted time-series design; ^l^ 12: if the intervention was conducted at a group level (e.g. a whole hospital, a community, etc.), statistical analysis took individual-level into account to determine effects at a group level; ^m^ NR: not reported; ^n^ NA: not applicable

**Supplementary Table 4.** Quality Assessment of controlled intervention studies (NIH Quality Assessment Scale)

| **Author** | **1^a^** | **2^b^** | **3^c^** | **4^d^** | **5^e^** | **6^f^** | **7^g^** | **8^h^** | **9^i^** | **10^j^** | **11^k^** | **12^l^** | **13^m^** | **14^n^** | **points** | **Quality** |
| --- | --- | --- | --- | --- | --- | --- | --- | --- | --- | --- | --- | --- | --- | --- | --- | --- |
| Brown et al. (2016) [8] | yes | yes | yes | NA^o^ | yes | yes | yes | yes | no | NR^p^ | yes | yes | yes | yes | 11 | Good |
| Brienza et al. (2018) [9] | yes | yes | NR | NA | yes | yes | yes | yes | NR | NR | yes | no | yes | no | 8 | Fair |
| Collins et al. (2018) [10] | yes | yes | yes | partly | no | yes | yes | yes | yes | NR | yes | yes | yes | yes | 11,5 | Good |
| Crist et al. (2021) [11] | yes | yes | NR | NA | NR | no | NR | NR | yes | NR | yes | yes | yes | yes | 7 | Fair |
| Crotty et al. (2019) [12] | yes | yes | yes | NA | yes | yes | no | yes | NR | yes | yes | yes | yes | yes | 11 | Good |
| Fairhall et al. (2012) [13] | yes | yes | Yes | NA | yes | yes | yes | NR | no | NR | yes | yes | yes | yes | 10 | Good |
| Hewitt et al. (2018) [14] | yes | yes | yes | NA | yes | yes | yes | yes | no | NR | yes | yes | yes | yes | 11 | Good |
| Hiyama et al. (2019)[15] | no | NA | no | NA | NR | yes | yes | yes | yes | NR | yes | NR | yes | yes | 7 | Fair |
| Jansen et al. (2018) [16] | yes | no | no | NA | NR | yes | yes | yes | yes | NR | yes | no | yes | yes | 7 | Fair |
| Kamga et al. (2017) [17] | yes | yes | yes | NA | yes | yes | yes | yes | no | NR | yes | yes | yes | yes | 11 | Good |
| Liddle et al. (2014) [18] | yes | yes | yes | NA | yes | yes | no | yes | NR | no | yes/no | yes/no | no | yes | 8 | Fair |
| Liu et al. (2021) [19] | yes | NR | NR | NA | yes | yes | no | NR | NR | NR | no | no | yes | yes | 5 | Low |
| Makizako et al. (2019) [20] | yes | yes | yes | NA | yes | yes | yes | yes | yes | NR | yes | yes | yes | yes | 12 | Good |
| Murabayashi et al. (2019) [21] | yes | yes | yes | NA | yes | yes | yes | yes | NR | no | yes | no | yes | no | 9 | Good |
| Siltanen et al. (2020) [22] | yes | yes | yes | NA | yes | yes | yes | yes | NR | NR | yes | yes | yes | yes | 11 | Good |
| Swan et al. (2019) [23] | yes | yes | yes | NA | no | yes | no | yes | NR | no | yes | no | yes | yes | 8 | Fair |
| Tanaka et al. (2021) [24] | yes | no | NR | NA | yes | yes | no | no | yes | yes | yes | no | yes | no | 7 | Fair |
| Tay et al. (2022) [25] | no | NA | no | NA | no | yes | no | NR | yes/no | NR | yes | no | yes | yes | 4.5 | Low |
| Uemura et al. (2021) [26] | yes | yes | yes | NA | yes | yes | yes | yes | NR | NR | yes | yes | yes | yes | 11 | Good |
| Ullrich et al. (2021) [27] | yes | yes | NR | yes | yes | yes | yes | yes | yes | NR | yes | NA | yes | NR | 10 | Good |

*Note*. ^a^ 1: randomized study; ^b^ 2: method of randomization adequate; ^c^ 3: treatment allocation concealed; ^d^ 4: participants and providers blinded; ^e^ 5: assessors blinded; ^f^ 6: groups similar at baseline on important characteristics; ^g^ 7: overall drop-out rate ≤ 20%; ^h^ 8: differential drop-out rate ≤ 15%; ^i^ 9: high adherence to the intervention protocols; ^j^ 10: other interventions avoided or similar; ^k^ 11: outcomes valid and reliable and implemented consistently; ^l^ 12: reported, that the sample size was sufficient for at least 80% power; ^m^ 13: outcomes reported or subgroups prespecified; ^n^ 14: intention-to-treat-analysis; ^o^ NA: not applicable; ^p^ NR: not reported

**References**

1. Jensen GL, Roy M-A, Buchanan AE, Berg MB. Weight loss intervention for obese older women: improvements in performance and function. Obes Res. 2004;12:1814–20.

2. Kamioka Y, Miura Y, Matsuda T, Iijima Y, Suzuki A, Nakazato K, et al. Changes in social participation and life-space mobility in newly enrolled home-based rehabilitation users over 6 months. J Phys Ther Sci. 2020;32:375–84.

3. Kato K, Yoshimi T, Aimoto K, Sato K, Itoh N, Kondo I. A rise-assisting robot extends life space and improves facial expressions of nursing home residents. BMC Health Serv Res. 2022;22:1588.

4. Levasseur M, Filiatrault J, Larivière N, Trépanier J, Lévesque M-H, Beaudry M, et al. Influence of Lifestyle Redesign(®) on Health, Social Participation, Leisure, and Mobility of Older French-Canadians. Am J Occup Ther Off Publ Am Occup Ther Assoc. 2019;73:7305205030p1–18.

5. Matsuda K, Ikeda S, Nakahara M, Ikeda T, Okamoto R, Kurosawa K, et al. Factors affecting the coefficient of variation of stride time of the elderly without falling history: a prospective study. J Phys Ther Sci. 2015;27:1087–90.

6. Nakagawa K, Inomata N, Konno Y, Nakazawa R, Hagiwara K, Sakamoto M. The Characteristic of a Simple Exercise Program under the Instruction of Physiotherapists-For General Elderly People and Frail Elderly People. J Phys Ther Sci. 2008;20:197–203.

7. Todo E, Higuchi Y, Ueda T, Murakami T, Kozuki W. A 3-month multicomponent home-based rehabilitation program for older people with restricted life-space mobility: a pilot study. J Phys Ther Sci. 2021;33:158–63.

8. Brown CJ, Foley KT, Lowman JDJ, MacLennan PA, Razjouyan J, Najafi B, et al. Comparison of Posthospitalization Function and Community Mobility in Hospital Mobility Program and Usual Care Patients: A Randomized Clinical Trial. JAMA Intern Med. 2016;176:921–7.

9. Brienza DM, Karg PE, Bertolet M, Schmeler M, Poojary-Mazzotta P, Vlachos H, et al. A Randomized Clinical Trial of Wheeled Mobility for Pressure Injury Prevention and Better Function. J Am Geriatr Soc. 2018;66:1752–9.

10. Collins KJ, Schrack JA, VanSwearingen JM, Glynn NW, Pospisil MC, Gant VE, et al. Randomized Controlled Trial of Exercise to Improve Walking Energetics in Older Adults. Innov aging. 2018;2:1–10.

11. Crist K, Jankowska MM, Schipperijn J, Rosenberg DE, Takemoto M, Zlatar ZZ, et al. Change in GPS-assessed walking locations following a cluster-randomized controlled physical activity trial in older adults , results from the MIPARC trial. Heal Place. 2021;69 April:102573.

12. Crotty M, Killington M, Liu E, Cameron ID, Kurrle S, Kaambwa B, et al. Should we provide outreach rehabilitation to very old people living in Nursing Care Facilities after a hip fracture? A randomised controlled trial. Age Ageing. 2019;48:373–80.

13. Fairhall N, Sherrington C, Kurrle SE, Lord SR, Lockwood K, Cameron ID. Effect of a multifactorial interdisciplinary intervention on mobility-related disability in frail older people: randomised controlled trial. BMC Med. 2012;10.

14. Hewitt J, Goodall S, Clemson L, Henwood T, Refshauge K. Progressive Resistance and Balance Training for Falls Prevention in Long-Term Residential Aged Care: A Cluster Randomized Trial of the Sunbeam Program. J Am Med Dir Assoc. 2018;19:361–9.

15. Hiyama Y, Kamitani T, Mori K. Effects of an Intervention to Improve Life-Space Mobility and Self-Efficacy in Patients following Total Knee Arthroplasty. J Knee Surg. 2019;32:966–71.

16. Jansen CP, Diegelmann M, Schilling OK, Werner C, Schnabel EL, Wahl HW, et al. Pushing the Boundaries: A Physical Activity Intervention Extends Sensor-Assessed Life-Space in Nursing Home Residents. Gerontologist. 2018;58:979–88.

17. Kamga H, McCusker J, Yaffe M, Sewitch M, Sussman T, Strumpf E, et al. Self-care tools to treat depressive symptoms in patients with age-related eye disease: a randomized controlled clinical trial. Clin Experiment Ophthalmol. 2017;45:371–8.

18. Liddle J, Haynes M, Pachana NA, Mitchell G, McKenna K, Gustafsson L. Effect of a Group Intervention to Promote Older Adults’ Adjustment to Driving Cessation on Community Mobility: A Randomized Controlled Trial. Gerontologist. 2014;54:409–22.

19. Liu M, Xue Q-LL, Gitlin LN, Wolff JL, Guralnik J, Leff B, et al. Disability Prevention Program Improves Life-Space and Falls Efficacy: A Randomized Controlled Trial. J Am Geriatr Soc. 2021;69:85–90.

20. Makizako H, Tsutsumimoto K, Doi T, Makino K, Nakakubo S, Liu-Ambrose T, et al. Exercise and Horticultural Programs for Older Adults with Depressive Symptoms and Memory Problems: A Randomized Controlled Trial. J Clin Med. 2019;9.

21. Murabayashi N, Akahoshi T, Ishimine R, Saji N, Takeda C, Nakayama H, et al. Effects of Music Therapy in Frail Elderlies: Controlled Crossover Study. Dement Geriatr Cogn Dis Extra. 2019;9:87–99.

22. Siltanen S, Portegijs E, Pynnönen K, Hassandra M, Rantalainen T, Karavirta L, et al. Effects of an Individualized Active Aging Counseling Intervention on Mobility and Physical Activity: Secondary Analyses of a Randomized Controlled Trial. J Aging Health. 2020;32:1316–24.

23. Swan F, English A, Allgar V, Hart SP, Johnson MJ. The Hand-Held Fan and the Calming Hand for People With Chronic Breathlessness: A Feasibility Trial. J Pain Symptom Manage. 2019;57:1051-1061.e1.

24. Tanaka S, Yamagami T, Yamaguchi H. Effects of a group-based physical and cognitive intervention on social activity and quality of life for elderly people with dementia in a geriatric health service facility: a quasi-randomised controlled trial. Psychogeriatrics. 2021;21:71–9.

25. Tay L, Tay EL, Mah SM, Latib A, Ng YS. Intrinsic capacity rather than intervention exposure influences reversal to robustness among prefrail community-dwelling older adults: A non-randomized controlled study of a multidomain exercise and nutrition intervention. Front Med. 2022;9.

26. Uemura K, Yamada M, Okamoto H. The Effectiveness of an Active Learning Program in Promoting a Healthy Lifestyle among Older Adults with Low Health Literacy: A Randomized Controlled Trial. Gerontology. 2021;67:25–35.

27. Ullrich P, Werner C, Bongartz M, Eckert T, Abel B, Schönstein A, et al. Increasing Life-Space Mobility in community-dwelling older persons with cognitive impairment following rehabilitation: A randomized controlled trial. J Gerontol A Biol Sci Med Sci. 2020. https://doi.org/10.1093/gerona/glaa254.
