# Supplementary material for: Multidimensional interventions to increase life-space mobility in older adults ranging from nursing home residents to community-dwelling: a systematic scoping review
Source: BMC Geriatr. 2023 Jul 6;23:412. doi: 10.1186/s12877-023-04118-3 (PMC10327334; doi:10.1186/s12877-023-04118-3)
Supplement: Supplementary file 4 — Supplementary Material 4 [file 12877_2023_4118_MOESM4_ESM.docx]

**Supplementary Table 5.** Overview intervention characteristics

| **Intervention category**  **level 1** | **Intervention category**  **level 2 + 3** | | **Study** | **Participants** | **Improvement in LSM** |
| --- | --- | --- | --- | --- | --- |
| Multidimensional interventions | Physical interventions + counseling | - | Brown et al. (2016) [1] | Hospitalized | 🗸 |
|  |  | - | Fairhall et al. (2012) [2] | Frail | 🗸 |
|  |  | - | Jensen et al. (2004) [3] | Community-dwelling | x |
|  |  | - | Kamioka et al. (2020) [4] | Patients in home-based rehabilitation | 🗸 |
|  |  | - | Tay et al. (2022) [5] | Prefrail | x |
|  |  | - | Todo et al. (2021) [6] | Patients in home-based rehabilitation | 🗸 |
|  |  | - | Ullrich et al. (2021) [7] | With cognitive impairment | 🗸 |
|  |  | + competence training of staff/relatives | Crotty et al. (2019) [8] | Nursing-home residents | 🗸 |
|  |  |  | Jansen et al. (2018) [9] | Nursing-home residents | 🗸 |
|  |  | + community improvements | Crist et al. (2021) [10] | Community-dwelling | 🗸 |
|  | Occupational therapy + home modifications | - | Liu et al. (2021) [11] | With restricted daily activities | 🗸 |
|  | Physical exercises + cognitive training/stimulation | - | Tanaka et al. (2021) [12] | Nursing-home residents (with and without dementia) | x |
| Purely physical interventions | Walking events/walking tasks |  | Hiyama et al. (2019) [13] | Patients with knee arthroplasty | 🗸 |
|  | Gait training + outdoor aerobic training |  | Collins et al. (2018) [14] | Community-dwelling | x |
|  | Physical exercises |  | Matsuda et al. (2015) [15] | Potentially in need of nursing care | 🗸 |
|  |  |  | Nakagawa et al. (2008) [16] | Frail | 🗸 |
|  |  |  |  | Non-frail | x |
|  |  |  | Hewitt et al. (2018) [17] | Nursing-home residents | x |
|  |  |  | Makizako et al. (2019) [18] | With depressive symptoms and memory problems | x |
| Purely counseling interventions | Promotion of behavioural changes towards a healthy lifestyle |  | Levasseur (2019) [19] | Community-dwelling (with and without disability) | 🗸/x |
|  |  |  | Uemura et al. (2021) [20] | Community-dwelling | 🗸 |
|  | Self-care tools + coaching |  | Kamga et al. (2017) [21] | With age-related eye diseases + depression | x |
|  | Suggesting out-of-home activities |  | Siltanen et al. (2020) [22] | Community-dwelling | x |
| Miscellaneous interventions | Breathing therapy + exercise advice + handheld fan + calming hand |  | Swan et al. (2019) [23] | With respiratory diseases | 🗸 |
|  | Driving cessation program |  | Liddle et al. (2014) [24] | With (planned) driving cessation | 🗸 |
|  | Rise-assisting robot |  | Kato et al. (2022) [25] | Nursing-home residents | 🗸 |
|  | Music therapy |  | Murabayashi et al. (2019) [26] | Frail | x |
|  | Wheelchair adaption |  | Brienza et al. (2018) [27] | Nursing-home residents | x |
|  | Horticulture activity program |  | Makizako et al. (2019) [18] | With depressive symptoms and memory problems | x |

**References**

1. Brown CJ, Foley KT, Lowman JDJ, MacLennan PA, Razjouyan J, Najafi B, et al. Comparison of Posthospitalization Function and Community Mobility in Hospital Mobility Program and Usual Care Patients: A Randomized Clinical Trial. JAMA Intern Med. 2016;176:921–7.

2. Fairhall N, Sherrington C, Kurrle SE, Lord SR, Lockwood K, Cameron ID. Effect of a multifactorial interdisciplinary intervention on mobility-related disability in frail older people: randomised controlled trial. BMC Med. 2012;10.

3. Jensen GL, Roy M-A, Buchanan AE, Berg MB. Weight loss intervention for obese older women: improvements in performance and function. Obes Res. 2004;12:1814–20.

4. Kamioka Y, Miura Y, Matsuda T, Iijima Y, Suzuki A, Nakazato K, et al. Changes in social participation and life-space mobility in newly enrolled home-based rehabilitation users over 6 months. J Phys Ther Sci. 2020;32:375–84.

5. Tay L, Tay EL, Mah SM, Latib A, Ng YS. Intrinsic capacity rather than intervention exposure influences reversal to robustness among prefrail community-dwelling older adults: A non-randomized controlled study of a multidomain exercise and nutrition intervention. Front Med. 2022;9.

6. Todo E, Higuchi Y, Ueda T, Murakami T, Kozuki W. A 3-month multicomponent home-based rehabilitation program for older people with restricted life-space mobility: a pilot study. J Phys Ther Sci. 2021;33:158–63.

7. Ullrich P, Werner C, Bongartz M, Eckert T, Abel B, Schönstein A, et al. Increasing Life-Space Mobility in Community-Dwelling Older Persons With Cognitive Impairment Following Rehabilitation: A Randomized Controlled Trial. J Gerontol A Biol Sci Med Sci. 2021;76:1988–96.

8. Crotty M, Killington M, Liu E, Cameron ID, Kurrle S, Kaambwa B, et al. Should we provide outreach rehabilitation to very old people living in Nursing Care Facilities after a hip fracture? A randomised controlled trial. Age Ageing. 2019;48:373–80.

9. Jansen CP, Diegelmann M, Schilling OK, Werner C, Schnabel EL, Wahl HW, et al. Pushing the Boundaries: A Physical Activity Intervention Extends Sensor-Assessed Life-Space in Nursing Home Residents. Gerontologist. 2018;58:979–88.

10. Crist K, Jankowska MM, Schipperijn J, Rosenberg DE, Takemoto M, Zlatar ZZ, et al. Change in GPS-assessed walking locations following a cluster-randomized controlled physical activity trial in older adults , results from the MIPARC trial. Heal Place. 2021;69 April:102573.

11. Liu M, Xue Q-LL, Gitlin LN, Wolff JL, Guralnik J, Leff B, et al. Disability Prevention Program Improves Life-Space and Falls Efficacy: A Randomized Controlled Trial. J Am Geriatr Soc. 2021;69:85–90.

12. Tanaka S, Yamagami T, Yamaguchi H. Effects of a group-based physical and cognitive intervention on social activity and quality of life for elderly people with dementia in a geriatric health service facility: a quasi-randomised controlled trial. Psychogeriatrics. 2021;21:71–9.

13. Hiyama Y, Kamitani T, Mori K. Effects of an Intervention to Improve Life-Space Mobility and Self-Efficacy in Patients following Total Knee Arthroplasty. J Knee Surg. 2019;32:966–71.

14. Collins KJ, Schrack JA, VanSwearingen JM, Glynn NW, Pospisil MC, Gant VE, et al. Randomized Controlled Trial of Exercise to Improve Walking Energetics in Older Adults. Innov aging. 2018;2:1–10.

15. Matsuda K, Ikeda S, Nakahara M, Ikeda T, Okamoto R, Kurosawa K, et al. Factors affecting the coefficient of variation of stride time of the elderly without falling history: a prospective study. J Phys Ther Sci. 2015;27:1087–90.

16. Nakagawa K, Inomata N, Konno Y, Nakazawa R, Hagiwara K, Sakamoto M. The Characteristic of a Simple Exercise Program under the Instruction of Physiotherapists-For General Elderly People and Frail Elderly People. J Phys Ther Sci. 2008;20:197–203.

17. Hewitt J, Goodall S, Clemson L, Henwood T, Refshauge K. Progressive Resistance and Balance Training for Falls Prevention in Long-Term Residential Aged Care: A Cluster Randomized Trial of the Sunbeam Program. J Am Med Dir Assoc. 2018;19:361–9.

18. Makizako H, Tsutsumimoto K, Doi T, Makino K, Nakakubo S, Liu-Ambrose T, et al. Exercise and Horticultural Programs for Older Adults with Depressive Symptoms and Memory Problems: A Randomized Controlled Trial. J Clin Med. 2019;9.

19. Levasseur M, Filiatrault J, Larivière N, Trépanier J, Lévesque M-H, Beaudry M, et al. Influence of Lifestyle Redesign(®) on Health, Social Participation, Leisure, and Mobility of Older French-Canadians. Am J Occup Ther Off Publ Am Occup Ther Assoc. 2019;73:7305205030p1–18.

20. Uemura K, Yamada M, Okamoto H. The Effectiveness of an Active Learning Program in Promoting a Healthy Lifestyle among Older Adults with Low Health Literacy: A Randomized Controlled Trial. Gerontology. 2021;67:25–35.

21. Kamga H, McCusker J, Yaffe M, Sewitch M, Sussman T, Strumpf E, et al. Self-care tools to treat depressive symptoms in patients with age-related eye disease: a randomized controlled clinical trial. Clin Experiment Ophthalmol. 2017;45:371–8.

22. Siltanen S, Portegijs E, Pynnönen K, Hassandra M, Rantalainen T, Karavirta L, et al. Effects of an Individualized Active Aging Counseling Intervention on Mobility and Physical Activity: Secondary Analyses of a Randomized Controlled Trial. J Aging Health. 2020;32:1316–24.

23. Swan F, English A, Allgar V, Hart SP, Johnson MJ. The Hand-Held Fan and the Calming Hand for People With Chronic Breathlessness: A Feasibility Trial. J Pain Symptom Manage. 2019;57:1051-1061.e1.

24. Liddle J, Haynes M, Pachana NA, Mitchell G, McKenna K, Gustafsson L. Effect of a Group Intervention to Promote Older Adults’ Adjustment to Driving Cessation on Community Mobility: A Randomized Controlled Trial. Gerontologist. 2014;54:409–22.

25. Kato K, Yoshimi T, Aimoto K, Sato K, Itoh N, Kondo I. A rise-assisting robot extends life space and improves facial expressions of nursing home residents. BMC Health Serv Res. 2022;22:1588.

26. Murabayashi N, Akahoshi T, Ishimine R, Saji N, Takeda C, Nakayama H, et al. Effects of Music Therapy in Frail Elderlies: Controlled Crossover Study. Dement Geriatr Cogn Dis Extra. 2019;9:87–99.

27. Brienza DM, Karg PE, Bertolet M, Schmeler M, Poojary-Mazzotta P, Vlachos H, et al. A Randomized Clinical Trial of Wheeled Mobility for Pressure Injury Prevention and Better Function. J Am Geriatr Soc. 2018;66:1752–9.
